# Repeat bleaching of a central Pacific coral reef over the past six decades (1960–2016)

**DOI:** 10.1038/s42003-018-0183-7

**Published:** 2018-11-08

**Authors:** Hannah C. Barkley, Anne L. Cohen, Nathaniel R. Mollica, Russell E. Brainard, Hanny E. Rivera, Thomas M. DeCarlo, George P. Lohmann, Elizabeth J. Drenkard, Alice E. Alpert, Charles W. Young, Bernardo Vargas-Ángel, Kevin C. Lino, Thomas A. Oliver, Kathryn R. Pietro, Victoria H. Luu

**Affiliations:** 1 Woods Hole Oceanographic Institution, Woods Hole, MA 02543 USA; 2 Massachusetts Institute of Technology-Woods Hole Oceanographic Institution Joint Program in Oceanography/Applied Ocean Science & Engineering, Woods Hole, MA 02543 USA; 3 Ecosystem Sciences Division, NOAA Pacific Islands Fisheries Science Center, Honolulu, HI 96818 USA; 4 Joint Institute for Marine and Atmospheric Research, University of Hawaii at Manoa, Honolulu, HI 96822 USA; 5 Princeton University, Princeton, NJ 08544 USA; 6 Ecosystem Sciences Division, NOAA Pacific Islands Fisheries Science Center, Honolulu, HI 96818 USA; 7 Joint Institute for Marine and Atmospheric Research, University of Hawaii at Manoa, Honolulu, HI 96822 USA; 8 Oceans Institute and School of Earth Sciences, The University of Western Australia, Crawley, 6009 Australia; 9 ARC Centre of Excellence for Coral Reef Studies, The University of Western Australia, Crawley, 6009 Australia; 10 Present Address: Scripps Institution of Oceanography, La Jolla, CA 92037 USA; 11 Present Address: United States Department of State, Washington, D.C. 20520 USA

## Abstract

The oceans are warming and coral reefs are bleaching with increased frequency and severity, fueling concerns for their survival through this century. Yet in the central equatorial Pacific, some of the world’s most productive reefs regularly experience extreme heat associated with El Niño. Here we use skeletal signatures preserved in long-lived corals on Jarvis Island to evaluate the coral community response to multiple successive heatwaves since 1960. By tracking skeletal stress band formation through the 2015-16 El Nino, which killed 95% of Jarvis corals, we validate their utility as proxies of bleaching severity and show that 2015-16 was not the first catastrophic bleaching event on Jarvis. Since 1960, eight severe (>30% bleaching) and two moderate (<30% bleaching) events occurred, each coinciding with El Niño. While the frequency and severity of bleaching on Jarvis did not increase over this time period, 2015–16 was unprecedented in magnitude. The trajectory of recovery of this historically resilient ecosystem will provide critical insights into the potential for coral reef resilience in a warming world.

## Abstract (web summary)

Hannah Barkley and Anne Cohen and colleagues used bleaching signatures in coral skeletons to examine the Jarvis Island coral community response to multiple El Niño heatwaves. They find the historically productive ecosystem experienced 10 bleaching events in the past 60 years and its recovery provides insights into coral reef resilience under ocean warming.

## Introduction

Rising ocean temperatures have had rapid, measurable, and devastating consequences for coral reefs worldwide^1^. Episodic thermal extremes superimposed on a secular warming trend are exposing coral communities to unfamiliar temperature regimes, triggering expulsion of the symbiotic zooxanthellae that supply organic carbon to their coral host. This process, termed bleaching, leads to starvation and often death, and bleaching-induced mortality has already caused extensive loss of global coral cover. While coral reefs can recover from severe bleaching, the time required for recovery is often long^2^. Consequently, climate model predictions of annual bleaching by 2050, if realized, are incompatible with the survival of most coral reefs through the end of this century^3^.

The 2015–16 El Niño caused sea surface temperature (SST) anomalies far in excess of those normally experienced by most coral reefs in the Pacific basin. For some, including the northern Great Barrier Reef, the 2015–16 event marked their first experience with severe thermal stress and led to widespread bleaching and mortality^4^. Conversely, coral reefs in the central equatorial Pacific, the epicenter of El Niño–Southern Oscillation dynamics, experience dramatic fluctuations in temperature every few years. Within the satellite SST era alone (1982–present), 3–4 °C temperature anomalies persisted across the region during three super El Niño’s, with 0.5–2 °C SST anomalies occurring every several years in between^5,6^. Co-occurring with these temperature changes are significant changes in ocean biogeochemistry, as wind-driven and topographic upwelling weaken or cease altogether, driving changes in upper ocean nutrient concentrations, primary productivity^7,8^, and carbonate system chemistry^9^.

**Fig. 1:**
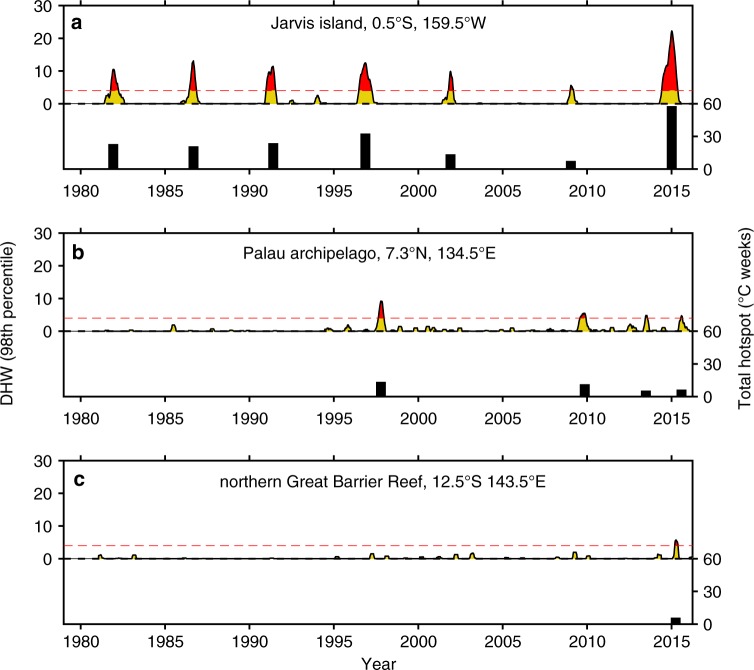
A comparative history of thermal stress represented by Degree Heating Weeks (DHWs) and cumulative DHWs or Total Hotspot on **a** Jarvis Island, central equatorial Pacific, **b** Palau, western tropical Pacific, and **c** northern Great Barrier Reef since 1980. DHWs > 4 (red dashed line) are considered conducive for coral bleaching and > 8 for severe bleaching and mortality. Jarvis corals experienced seven > 4 and six > 8 DHW episodes since 1980. Palau corals experienced two episodes > 4 DHW (1998 and 2010). For corals in the northern GBR, the 2016 thermal anomaly was their first encounter with ocean conditions considered conducive for bleaching. Here, DHWs are calculated using a percentile method rather than the traditional mean monthly maximum (MMM) to estimate maximum mean SST experienced by each reef. This approach was taken to enable direct comparison between regions dominated by inter-annual SST variability (central equatorial Pacific) and those dominated by seasonal SST variability (western tropical Pacific). A detailed description of the percentile method and the comparison with traditional NOAA DHWs for these three sites are provided in the Methods and Supplementary Information

Such environmental extremes are felt strongly by the coral communities of Jarvis Island, an uninhabited coral reef ecosystem within the US Pacific Remote Islands Marine National Monument (0.37°S, 159.99°W). Here, degree heating weeks (DHW), a metric of accumulated temperature stress^10^, approached and/or exceeded 10 °C-weeks six times since 1982 (Fig. 1). While such conditions are considered conducive for repeat episodes of catastrophic bleaching and mortality, Jarvis appears to have remained highly productive over much of this time. Surveys conducted between 2000 and 2009 revealed total cover of reef-building organisms (primarily corals and coralline algae) close to 50%, exceeding the central Pacific average for uninhabited islands, and turf and macroalgal cover significantly lower than average^11^. Further, fish populations on Jarvis are dominated by the highest trophic levels, and represent one of the largest concentrations of fish biomass for coral reefs in the central and western Pacific^12^. These observations have raised questions about the nature of the response of the Jarvis coral communities, and others located in the central equatorial Pacific, to repeated exposure to extreme conditions at frequencies expected to devastate most tropical reefs by mid-century^13^.

Here we present a history of the Jarvis coral community response to repeat El Niño-induced heatwaves spanning the last six decades, reconstructed using a bleaching proxy archived in the skeletons of massive corals that survived these events. Stress bands, anomalously high-density bands revealed in the skeletons of massive, long-lived corals by x-radiography or 3-D computerized tomography (CT) scanning, have long been qualitatively associated with coral bleaching^14–18^. Recently, Barkley and Cohen^19^ demonstrated a strong correlation between the proportion of stress bands in populations of massive *Porites* corals in Palau and the observed severity of community-wide bleaching at eleven lagoon, patch, and barrier reef stations during the 1997–8 and 2009–10 El Niño’s. This finding provided a quantitative tool with which to evaluate the severity of the coral reef response to historical thermal stress in the absence of real-time visual observations.

In this study, we use ecological surveys conducted on Jarvis in November 2015 during the peak of the 2015–16 El Niño, and data from Howland Island in 2010, to validate the use of the skeletal bleaching proxy outside of Palau. Skeletal cores extracted from massive Jarvis *Porites* corals before (2010, 2012), during (2015) and after (2016, 2017) the bleaching event allow us, for the first time to our knowledge, to link stress band formation with active bleaching, to evaluate the underlying mechanism for stress band formation, and to track the incorporation of the bleaching signal into the growing skeleton. Finally, we use stress bands archived in the skeletons of long-lived coral survivors to reconstruct a quantitative history of bleaching on Jarvis and to place the severity of the 2015–16 event on Jarvis in the context of the last six decades.

## Results

### The 2015–16 coral bleaching event at Jarvis

**Fig. 2:**
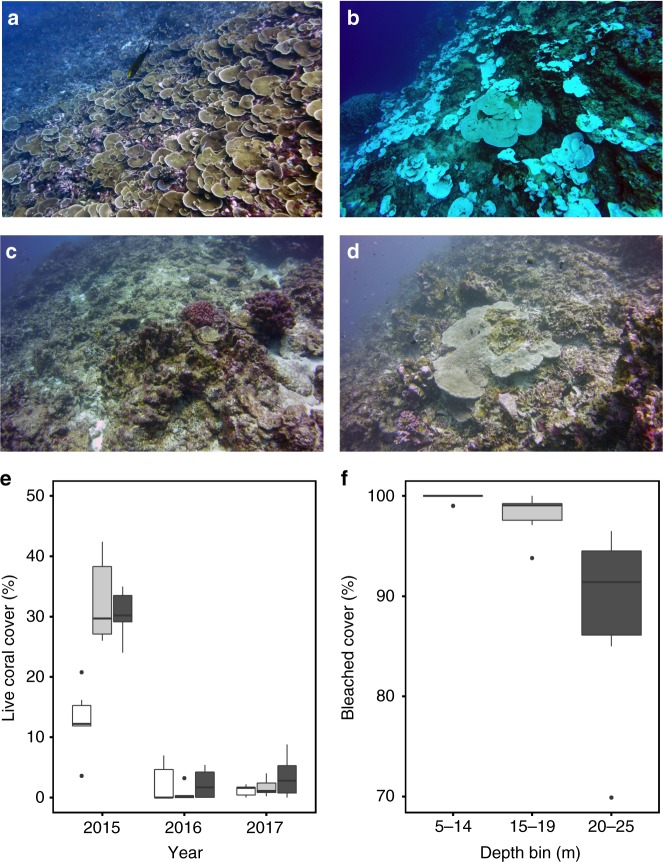
The impact of the 2015 El Niño on Jarvis Island coral communities recorded by the first visual surveys of the reef during and after peak El Niño. **a** *Montipora*-dominated reef communities in April 2015 (NOAA/Paula Ayotte). **b** The same communities ~100% bleached but still alive in November 2015. **c** In May 2016, ~95% of corals were dead. **d** By April 2017, some *Porites* corals had recovered while cover remained low. **e** Mean ( ± standard error) live coral cover in November 2015 (the sum of living bleached and healthy non-bleached colonies), May 2016 and Apr 2017. No bleaching was observed in 2016 and 2017. Depths are 5–14 m (white), 15–19 m (light gray), and 20–25 m (dark gray). **f** Mean ( ± standard error) of coral cover bleached in Nov 2015 at three depth bins

Multiple ecological surveys of Jarvis have been conducted by the Ecosystem Sciences Division of the National Oceanic and Atmospheric Administration (NOAA) Pacific Islands Fisheries Science Center since 2000^20^. However, prior to 2015, none of these surveys coincided with peak El Niño conditions. Minor bleaching (~3%) was recorded in April 2010, although those surveys occurred after the El Niño had subsided^21^. We conducted an expedition to Jarvis Island from 13^th^ to 16^th^ November 2015 coinciding with the peak SST anomaly associated with the 2015–16 El Niño. Our expedition provided the first opportunity to directly observe and measure the reef response to extreme heat. At the time of our arrival on site, SST anomalies in the region had exceeded 3 °C for 20 consecutive weeks (Fig. 1, Supplementary Figures 1–2). Photographic surveys conducted along triplicate 50 m transects spanning 5 m to 25 m depth revealed average live coral cover of 25.3% cover (±2.5% SE) with visible bleaching in 95.4% (±1.8% SE) of coral-covered substrate, and a small but significant decrease with depth (two-way ANOVA, F_2,14_ = 6.64, *p* = 0.009) (Fig. 2, Supplementary Tables 1–2). Levels of bleaching near 100% were observed in fast-growing *Montipora* colonies dominant on the island’s western (leeward) side, branching *Pocillopora* colonies abundant on the island’s eastern (windward) side, and massive *Porites* colonies, some exceeding 100 years in age. Discrete water samples and instrument deployments documented a dramatic shift in nearshore chemistry concurrent with elevated temperatures. Nitrate concentrations on the reef decreased from the climatological mean of 5 µM^22^ to levels at or below detection. Both pH and aragonite saturation state (Ω_ar_) increased above climatology^22^, likely due to a combination of upwelling cessation and a reduction in reef calcification (Supplementary Figures 3–4, Supplementary Table 3).

**Fig. 3:**
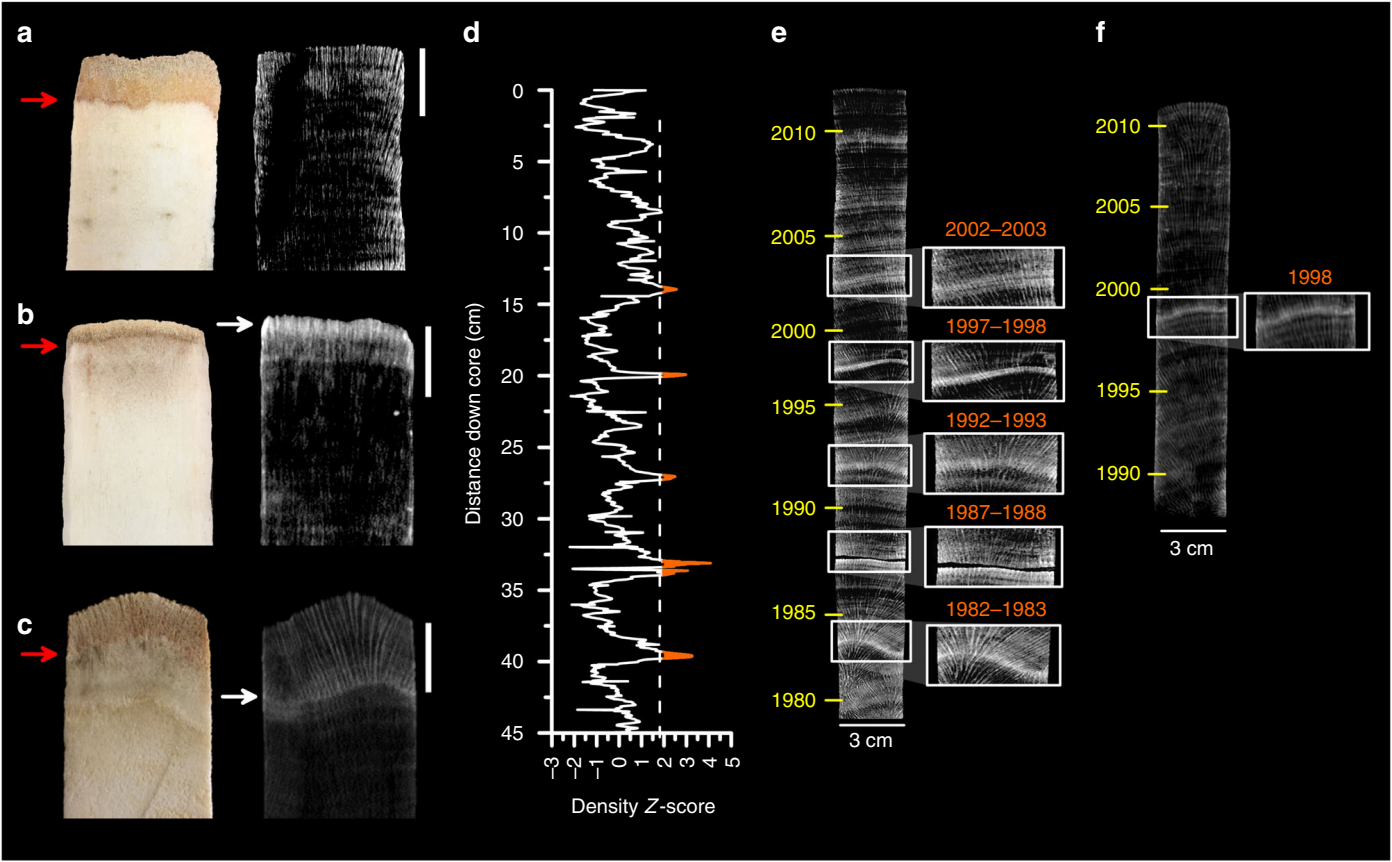
The skeletons of *Porites* corals record the 2015 bleaching (**a**–**c**) and historical bleaching events (**d**–**e**) that occurred on Jarvis Island, and for comparison, on Palau in 1998 (**f**). In the CT scan images, white is high-density, black is low density. In (**a**) CT scans of the tops of skeletal cores extracted from massive Jarvis *Porites* in 2012 show no sign of stress band formation. 2012 was an El Niño–Southern Oscillation neutral year and average tissue thickness measured in the core tops (left, red arrow) approached 1 cm, indicating the corals were energetically replete. Conversely, in (**b**), CT scans of 88% of the cores extracted in November 2015 revealed high-density stress band in the process of formation (white arrow). Tissue thickness of bleached colonies was reduced by ~75% (red arrow) indicating starvation. In (**c**), a core removed in April 2017 reveals recovery of tissue thickness (red arrow) and the 2015 stress band sequestered beneath new skeletal growth (scale bar = 1 cm). Stress bands are confirmed by automated analysis of density variations (**d**) where Z-scores > 2 (dashed line) in the detrended density time series are considered stress bands. Long *Porites* cores from Jarvis reveal multiple historical stress bands (**e**) whereas those from Palau have one or two, consistent with documented bleaching events on Palau in 1998 and 2010 (**f**)

Skeletal cores extracted in November 2015 from bleached *Porites* colonies revealed the impact of prolonged bleaching on skeletal growth. In 3-D Computerized Tomography (CT) scans, unusually high-density bands (called stress bands), otherwise invisible to the naked eye, were observed forming at the top of 88% of the cores (Fig. 3a, b). In addition, the cores revealed an ~50% decrease in tissue thickness, from an average ( ± SE) of 8.3 mm ( ± 0.4 mm) in colonies we had sampled during neutral periods of the El Niño–Southern Oscillation (September 2012 and April 2010) to 4.8 mm (±0.4 mm) in colonies we sampled in November 2015 (Two-sided Welch *T*-test, *t* = 6.3, df = 33.9, 95% CI = 2.3, 4.6, *p* < 0.001). The decrease in tissue thickness, which likely reflects the bleached corals’ metabolism of their own biomass to fuel basic physiological functioning during starvation^17,23^, plays a major role in stress band formation by limiting the ability of the coral to extend its skeleton upward during calcification^18^. Consequently, instead of using newly accreted calcium carbonate to extend upward, the bleached coral thickens existing skeleton, resulting in a discrete, anomalously high-density band visible in the CT image.

**Fig. 4:**
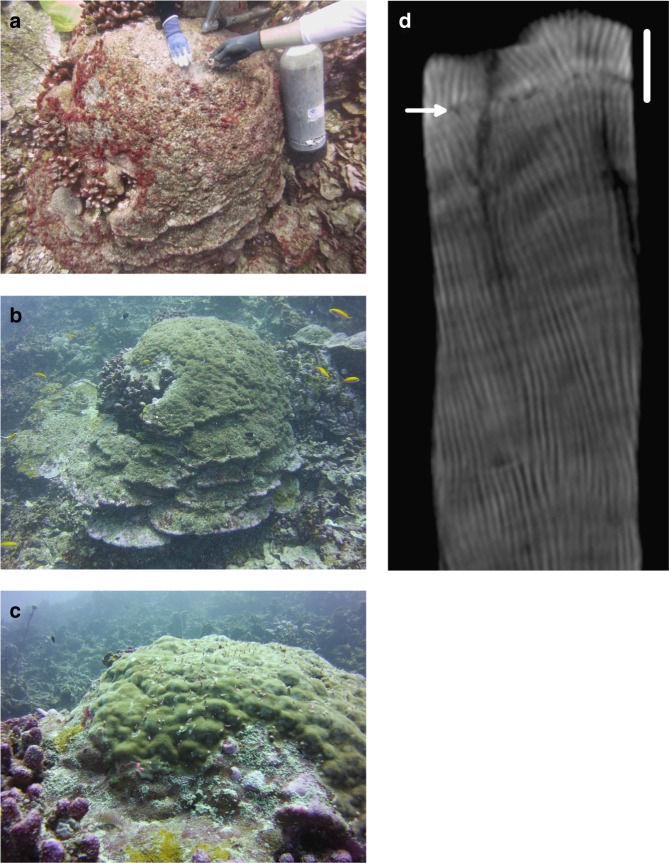
Bleaching, tissue loss and recovery of a massive *Porites* coral on Jarvis Island, and incorporation of the bleaching signal into the skeleton. **a** *Porites* ID 497 at 16.5 m depth on the west side of the island (0.369 °S, 160.008 °W) bleached in 2015, and in May 2016, no live tissue was evident on the colony surface. **b**, **c** By April 2017, the coral exhibited almost full recovery. In (**d**) A 3-D CT scan of a core removed from the recovered colony in 2017 revealed almost 1 cm of new growth above the stress band, a growth rate ~30% lower than pre-bleaching rates (scale bar = 1 cm). A mortality scar (arrow), signaling complete localized loss of tissue for an extended period, is also visible in the scan. Corallite tracks, which are the skeletons of individual polyps, are continuous across the mortality scar, indicating some polyps survived the bleaching deep inside the skeleton, revived and continued to extend their original corallites once ocean conditions returned to normal

The prolonged 2015–16 bleaching event on Jarvis led to severe coral mortality (Fig. 2). Low temperature spikes recorded by in situ temperature loggers deployed on the west side of the island, as well as elevated concentrations of dissolved inorganic nutrients in discrete water samples and decreases in both pH and Ω_ar_, revealed that upwelling had resumed by the time of our follow-up expedition in May 2016 (Supplementary Figures 2–4). However, live coral cover had plummeted from 25.3% ( ± 2.5% SE) to 1.7% ( ± 0.6% SE) (Three-way ANOVA with post hoc Tukey HSD; 2015-2016: diff = −23.6%, 95% CI = -28.3, −18.9, *p* < 0.001; Supplementary Tables 1-2). Along our survey transects, evaluated in both 2015 and in 2016, mortality of non-massive genera, including previously dominant *Montipora* and *Pocillopora* corals, was nearly 100%, consistent with results of independent island-wide surveys^20^. Amongst the surviving corals, we observed colonies of *Acropora*, *Hydnophora*, *Pavona*, and *Favia* spp., as well as massive *Porites* which exhibited extensive partial mortality (Fig. 4).

We returned to Jarvis Island again in April 2017, 18 months after peak bleaching. Coral cover had not measurably increased (Fig. 2), but initial signs of recovery were evident. Live juvenile colonies of eleven Scleractinian genera, including *Pocillopora*, *Porites*, *Leptoseris, Favia*, and *Psammocora* were observed and counted (Supplementary Figure 5, Supplementary Table 4) and crustose coralline algae (CCA) cover, several millimeters thick, had regained pre-bleaching levels (Supplementary Figure 6, Supplementary Table 5). Several massive *Porites* colonies that appeared dead with no sign of living polyps in 2016 were once again covered with healthy zooxanthellate-laden tissue, and tissue thickness had recovered to pre-bleaching levels (average 8.21 mm ± 0.14, *n* = 12) (Figs. 3c, 4). CT scans of skeletal cores extracted from the recovered colonies in 2017 revealed the 2015 stress bands and mortality scars now entrapped beneath a new layer of skeletal growth (Figs. 3c, 4). In some colonies, post-2015 growth appeared to have been initiated by the same polyps that created the stress band but were presumed dead from the prolonged bleaching. In these colonies, the individual corallite tracks of these polyps are traceable from beneath and across the stress band and into the post-bleaching skeletal growth (Fig. 4d).

### Skeletal reconstructions of historical coral bleaching

**Fig. 5:**
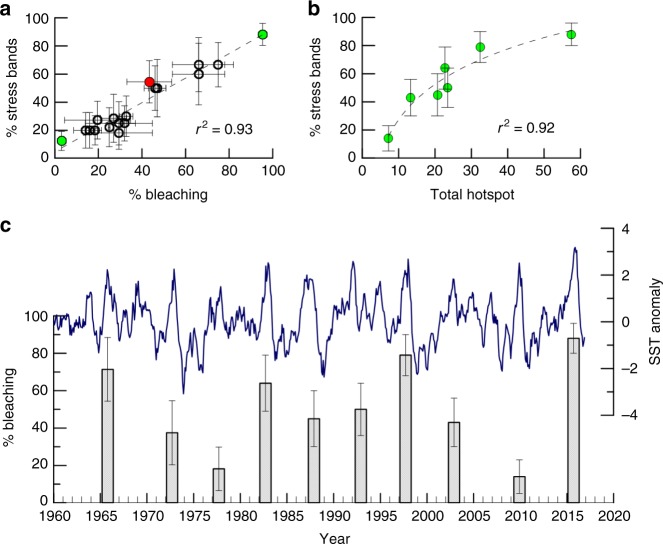
Historical coral reef bleaching events on Jarvis Island reconstructed from stress bands. In (**a**) percent of *Porites* corals with a stress band in a given year is strongly correlated with the observed level of community bleaching in the same year ( ± one standard error) for Jarvis 2015 (this study) and 2010 (green)^21^, Howland 2010 (red)^21^, and Palau 1998 and 2010 (open black circles)^19^. This relationship allows us to reconstruct the history of bleaching on Jarvis in the absence of visual observations. **b** Percent of Jarvis *Porites* corals with a stress band in a given year scale with the level of thermal stress experienced by the community that year, indicating that the Jarvis coral community responds predictably to thermal stress. Total Hotspot, an index of the cumulative thermal stress during a specific event, is calculated from weekly satellite SST spanning the time period 1982–2016. In (**c**) six decades of coral reef bleaching on Jarvis Island (vertical bars, mean ± one standard error), 1960–2016, constructed from stress bands using the calibration in (**a**). The time series of ERSST anomalies over the same time period, for a 2° × 2° grid centered on Jarvis, is shown in blue. All bleaching events coincide with high SST anomalies

Visual surveys during three separate expeditions during and after the 2015–16 El Niño recorded catastrophic bleaching and mortality on Jarvis in response to extreme and prolonged heat. Further, massive, long-lived *Porites* corals that bleached, starved, lost tissue mass, and subsequently recovered, archived a record of the bleaching event as anomalously high-density stress bands within their skeletons (Fig. 3). The proportion of *Porites* colonies presenting with 2015–16 stress bands was consistent with the catastrophic scale of bleaching and mortality in the Jarvis coral community (Fig. 5a).

However, examination of the CT scans of longer skeletal cores extracted from Jarvis *Porites* indicate that 2015–16 was not the first time these corals had formed stress bands. Indeed, multiple stress bands are apparent down the length of the majority of the cores (Fig. 3e, Supplementary Figure 7). We used annual high-low density band counts combined with annual extension rates estimated by the distance between successive monthly dissepiments^18^ to assign ages to all the historical stress bands. Two cores extend back to the turn of the 20^th^ century, and the earliest stress bands appear in these cores in 1912, indicating that bleaching occurred on Jarvis over 100 years ago. However, the error on the stress band proportions derived from only two cores was too large to support a meaningful interpretation of bleaching severity in the context of the observational data. Therefore, in this study, we quantified stress band proportions—the fraction of coral cores with a stress band in a given year relative to the total number of cores examined—and reconstructed a history of beaching severity for the period 1960–2016, with a minimum of 7 cores represented in each year (Supplementary Tables 6–7).

Between 1960 and 2016, we identified 10 episodes of stress band formation, always occurring during documented historical El Niño events and periods of prolonged elevated SSTs. To evaluate the degree of stress band formation relative to levels of thermal stress imposed on the Jarvis coral community during each event, we calculated (DHW) and the cumulative or Total Hotspot (i.e., the total number of weeks exceeding 1 °C above the mean maximum SST)^10^, using the weekly resolved satellite-derived OISST data product (https://www.ncdc.noaa.gov/oisst). Because satellite-derived weekly SSTs are only available from 1982 through to present, our DHW and Hotspot calculations are restricted to the period 1982–2016 (Fig. 1, Supplementary Figure 1). Additionally, conventional NOAA DHW and Hotspot calculations were developed for off-equatorial regions, where SST variability is dominated by the seasonal cycle but are inappropriate for the central equatorial Pacific, where SSTs are dominated by inter-annual variability. Thus, we used a percentile method rather than the traditional mean monthly maximum to estimate the maximum mean SST used in the DHW and Hotspot calculations (see Methods for a detailed description of the percentile method). From 1982–2016, the proportion of *Porites* corals that formed stress bands during each heatwave was highly correlated with the degree of thermal stress imposed (Pearson’s r^2^ = 0.93; *p* < 0.001) (Fig. 5b), with no evidence for acclimatization. Indeed, the greater the level of thermal stress, the higher the number of *Porites* corals on Jarvis formed a stress band that year.

We then derived a stress band–community-level bleaching calibration by regressing *Porites* stress band proportions against observational bleaching data for all coral genera collected by ecological surveys conducted on three Pacific reefs: Jarvis in 2015 (this study) and 2010^21^, Howland Island in 2010^21^ (0.8113 °N, 176.6183 °W) and Palau in 1998 and 2010^19^ (7.5150 °N, 134.5825 °E). The proportion of stress bands in the *Porites* populations and the percent bleaching observed in the coral reef community in a given year are strongly correlated (% bleaching = 1.07 ± 0.07 SE (% stress bands)—2.50 ± 3.16 SE; Pearson’s r^2^ = 0.93; *p* < 0.001) (Fig. 5a). We applied the calibration to the full historical stress band record from Jarvis to reconstruct the record of community-wide bleaching at Jarvis back through 1960. Our results reveal a history of repeat bleaching including moderate (<30% bleaching) and severe (>30% bleaching) events, in (mean ± SE) 2015–16 (91% ± 6%), 2009–10 (12% ± 7%), 1997–98 (82% ± 9%), 2001–02, 1992–93, and 1987–88 (40–50% ± 11–14%), 1982–83 (66% ± 14%), 1977–78 (17% ± 10%), 1972–73 (38% ± 16%), and 1965–66 (74% ± 16%) (Fig. 5c).

The Extended Reconstructed Sea Surface Temperature (ERSST) data product (https://iridl.ldeo.columbia.edu/SOURCES/.NOAA/.NCDC/.ERSST/.version3b) provides monthly resolved SST estimates in a 2° × 2° grid box centered on Jarvis Island. We used this data product to evaluate the magnitude of the 2015–16 SST anomaly on Jarvis in the context of the historical record of SST at this site, 1960–2016. The magnitude of the 2015–16 thermal anomaly on Jarvis was unprecedented since 1960 (Fig. 5c), and our historical bleaching reconstruction reveals that the severity of community-wide bleaching was similarly exceptional. However, contrary to the global trend^24^, we do not detect a statistically significant increase in the severity of bleaching over time nor an increase in the frequency of bleaching events in the last 60 years (Pearson’s r^2^ = 0.013, *p* = 0.74). The severity of bleaching in 1997–98, 1982–83, and 1965–66 were statistically within one standard error of 2015–16, suggesting that Jarvis has experienced one episode of catastrophic bleaching every 15 years on average, since 1960. If all bleaching events are considered, the record implies that the Jarvis coral community has bleached with varying degrees of severity every five years, on average. Despite a long history of repetitive bleaching, Jarvis was ranked one of the healthiest ecosystems in the global ocean in 2012^25^. Taken together, these observations suggest uncommon resilience of a coral reef community exposed to repeated, dramatic fluctuations in ocean temperature and biogeochemical change.

### Recovery potential of Jarvis coral reefs

Under certain circumstances, coral reefs are able to recover from catastrophic bleaching-induced mortality, but require time to do so^26–28^. Thus, concerns about coral reef futures under 21^st^ century ocean warming are centered primarily around high-frequency, repeat bleaching events which may prevent coral communities from achieving full recovery before bleaching occurs again and may preclude adaptation^24^. Our historical bleaching reconstruction reveals a coral reef community that has bleached frequently, and at times catastrophically, yet appears to have maintained a healthy state over time. Understanding the mechanisms underlying such resilience could provide key insights into the conditions under which reefs might tolerate 21^st^ century ocean warming and help to advance successful management strategies under global climate change.

Our data suggest that Jarvis corals are not resistant to thermal stress, as are some coral reef communities considered to be climate refugia^29^. Indeed, our bleaching record indicates that the severity of bleaching of the Jarvis coral community has been proportional to the level of thermal stress imposed over the last six decades. Furthermore, our skeletal records show that Jarvis corals bleach repeatedly and, based on our observations of the impact of the 2015–16 heatwave, it is likely that many probably die during the most extreme events. Enhanced productivity of the central equatorial Pacific, fueled by trade wind and topographic upwelling^30^, as well as the remote location of the Jarvis system relative to human populations^31^, may play key roles in its recovery. On Jarvis, extreme El Niño events that cause bleaching are generally followed by an abrupt resumption of upwelling during which cool, nutrient-rich waters fuel rapid tissue biomass renewal in some species (Fig. 4, Supplementary Figures 2–4). Jarvis hosts dense populations of herbivorous fish^12^ that prevent fast-growing macroalgae from overgrowing the reef substrate^13,32^, and likely stall the shift from coral to algal dominated systems as occurred in the Caribbean^33^. In 2016 and 2017, we observed rapid re-establishment of CCA on Jarvis, as seen in the Phoenix Islands after the 2002–2003 bleaching^34^, likely enabled by effective grazing and resumption of upwelling (Supplementary Figure 6, Supplementary Table 5). This step in coral community recovery is critical because CCA helps to stabilize the substrate following coral mortality and is a favored settlement substrate for coral larvae^35^ (Supplementary Figure 5, Supplementary Table 4). Finally, *Montipora* and *Pocillopora* species, abundant on Jarvis prior to 2015, are fast-growing coral genera capable of quickly recolonizing a devastated reef following successful recruitment^36,37^. Indeed the dominance of these genera in Jarvis’ highly productive, albeit relatively depauperate, coral community may be a strategic trade-off that enhances the resilience of coral reefs in highly stressful environments.

With less than 5% live corals remaining in 2017, it is uncertain whether new recruits are being supplied primarily by the few survivors, from deeper dwelling corals that may have been unaffected by the bleaching, or by larvae from neighboring islands. The magnitude and duration of the 2015 heat stress was likely the highest and longest that Jarvis Island coral communities have ever experienced, a fact that will likely prolong its recovery relative to prior years. Yet, the historical record implies that Jarvis has recovered from catastrophic events in the past and gives reason to hope that Jarvis will regain its previously vibrant and productive coral-based ecosystem. Further, the protected status of Jarvis in the Pacific Remote Islands Marine National Monument ensures intact populations of grazers, and eliminates land-based sources of sediment and pollution, additional safeguards that are known to maximize the chances of recovery.

## Conclusions

The coral communities on Jarvis Island, a highly productive coral reef ecosystem in the central equatorial Pacific, experienced catastrophic bleaching and mortality during the 2015–16 El Niño. Massive long-lived *Porites* corals that bleached, starved, suffered extensive partial mortality, and recovered from the prolonged heatwave, archived a record of the reef-wide bleaching event as discrete high-density stress bands within their skeletons. In this study, we showed that the proportion of stress bands in populations of *Porites* corals sampled on three reef systems including Jarvis, scales with the severity of bleaching in the coral communities as recorded by visual observations. Applying this relationship to down-core records of Jarvis *Porites* stress bands reveals that multiple historical bleaching events, three of them catastrophic, occurred on Jarvis Island between 1960 and 2016. We found that the frequency and severity of bleaching events did not increase over this time period. Nevertheless, the magnitude of the 2015–16 thermal anomaly at Jarvis and the severity of the 2015–16 bleaching were unprecedented in the record. We believe that the timing and trajectory of recovery of this historically resilient ecosystem will provide critical new insights into the potential for coral reef survival in an era of unprecedented ocean change.

## Methods

### Percentile-based method for calculated thermal stress

The traditional DHW calculation (1 °C above the Maximum Monthly Mean or MMM) cannot be meaningfully applied to regions dominated by inter-annual SST variability, prohibiting a comparison of thermal stress on a global scale. The MMM is average temperature of the warmest month over several pre-specified years. However in the central equatorial Pacific, peak temperatures do not occur during the same month in every year and the traditional MMM calculation generally underestimates the high end of temperatures that corals normally see at these sites. Consequently, central equatorial Pacific DHWs calculated using the traditional method are generally overestimated (Supplementary Figure 1). To enable direct comparison of thermal stress on reefs dominated by seasonal- vs. inter-annual SST variability, a percentile-based thermal threshold was developed to estimate the maximum temperature corals normally experience. Average weekly satellite-based SSTs (IGOSS Reyn_Smith OIv2, 1° × 1° resolution, https://iridl.ldeo.columbia.edu/SOURCES/.IGOSS/.nmc/.Reyn_SmithOIv2) during neutral years of the El Niño–Southern Oscillation (1984–5, 1990, 1993, 1996) are binned by percentile at increments of 0.01 percentile. A test was applied to determine which percentile best predicts observed bleaching when used as the bleaching threshold (i.e., equivalent to the MMM + 1 in the DHW calculation). DHWs were calculated for all coral reef sites reported in Donner et al., (2017)^38^, using bleaching thresholds estimated across a range of threshold percentiles (90.00^th^ to 99.99^th^). The derived DHWs were compared against the database of bleaching observations^38^, considering each year at each site an individual event *i* for a total of *n* = 21,384 predictions. Bleaching was predicted for each *i* if the DHW exceeded 4 °C-weeks during that year at that site, consistent with the traditional DHW “bleaching likely” prediction. The overall quality of prediction was assessed using a Brier Score (Supplementary Figure 8). The power of the model to predict actual bleaching was maximum at the 98^th^ percentile. In a comparison of the percentile-based model with the traditional MMM model, the percentile-based model outperformed the traditional MMM in both Brier Score and predictive power, while maintaining a similar number of Type 1 errors (Supplementary Table 8). Analysis of temperature time series was conducted in MATLAB (2017a).

### Field expeditions and Permits

Coral skeletal cores, ecological survey data, seawater samples, and in situ instrument time series were collected during seven expeditions to Jarvis Island between 2008 and 2017, aboard the NOAA ship Hi’ialakai (27–29 March 2008, 2–4 April 2010, 3–5 May 2012, 2–5 April 2017), Pangaea Exploration S/V Sea Dragon (13–16 September 2012), R/V Machias (12–15 November 2015), and NOAA ship Oscar Elton Sette (17–23 May 2016). Research activities and sample collection were conducted under U.S. Fish and Wildlife Service Pacific Reefs National Wildlife Refuge Complex Research and Monitoring Special Use Permits 12521-10001 (effective date: 15 Jan 2010; expiration date: 30 May 2010), 12521-12001 (effective date: 7 Feb 2012; expiration date: 31 Dec 2012), 12521-12005 (effective date: 29 Aug 2012; expiration date: 30 June 2014), 12521-14001 (effective date: 1 Jan 2015; expiration date: 31 Dec 2015), and 12513-15001 (effective date: 11 Nov 2015; expiration date: 31 Dec 2015) and in compliance with Presidential Proclamation 8336.

### Coral skeletal core collection and analysis

Skeletal cores were collected from *Porites* coral colonies in April 2010 (*n* = 4), May 2012 (*n* = 3), September 2012 (*n* = 6), November 2015 (*n* = 16), May 2016 (*n* = 1) and April 2017 (*n = 1*, used here for imaging purposes only). All cores were collected from colonies at 3–17 m depth using pneumatic or hydraulic drills with diamond drill bits (Supplementary Table 6). Cores collected in 2010 and 2012 were sampled from healthy colonies and were between 50 and 200 cm in length. In 2015, cores were collected from bleached *Porites* colonies, and were limited to 5–10 cm length in accordance with United States Fish and Wildlife Service permitting restrictions. The core collected in May 2016 was collected from a recently dead portion of a massive colony that experienced substantial tissue mortality during the 2015–16 bleaching event. The core colleced in 2017 was extracted from a recovered colony. Core holes left in the coral colonies were filled with cement plugs, sealed with underwater epoxy, and secured flush with the existing colony surface. Visual inspections of coral colonies several years after coring demonstrated full recovery and complete tissue overgrowth of the cement plug.

Coral cores were oven-dried and scanned with a Siemens Volume Zoom Helical Computerized Tomography (CT) Scanner at WHOI and at the University of North Carolina Biomedical Research Imaging Facility. Density banding and stress band presence was evaluated in 3-D CT scans of coral cores using the automated *coralCT* software^39^. Density time series were extracted and averaged from individual polyp growth tracks, which accounts for the different ages of skeleton in horizontal cross sections due to uneven growth geometry, in 0.1 mm increments from the top of the skeletal core up to 70 cm down core. Density values were converted to Z-scores by subtracting the long-term core mean density from each raw density value and dividing by the long-term standard deviation. High-density stress bands were defined as bands greater than 1 mm thick that spread across the entire width of the core where density values exceeded two standard deviations of the whole core density mean (i.e., a Z-score greater than 2). Stress bands that formed prior to 2010 were identified based on density banding patterns counted downward from the core top. Stress bands that were forming in 2015–16 were dated based on their location at the very top of the core (indicating that they were forming during the time of collection). Coral tissue thickness, measured as the vertical distance between the top of the core to the most recently accreted dissepiment, was measured on a slice of skeleton cut from the top of each core using a Nikon SMZ1500 stereomicroscope and SPOT imaging software.

### Ecological surveys

Repeat transect surveys were conducted at Jarvis during the height of the bleaching event (November 2015), at six months (May 2016), and again at sixteen months (April 2017) post-bleaching. Three 50 m surveyed at each of three depths (shallow: 5–14 m, mid-depth: 15–19 m, and deep: 20–25 m) on the west (all depths: 0.369 °S, 160.008 °W) and east sides (shallow: 0.374 °S, 159.983 °W, mid and deep: 0.367 °S, 159.979 °W) of the island. Each replicate 50 m transect was laid ~5 m apart in the cross-shore direction, and a photograph of a 0.5 m × 0.5 m quadrat taken every meter. Photographs were analyzed using Coral Point Count with Excel extensions^40^. Live coral cover of each photograph was evaluated by randomly overlaying ten points on each image and identifying the type of substrate (coral vs. non-coral) and any coral colony to the genus level, with 500 points identified per transect and 1500 points identified per depth. In 2015, random points that fell on live coral were identified as healthy (pigmented tissue) or bleached (non-pigmented living tissue), with the bleached cover calculated as the total number of random points located on bleached tissue divided by the total number of points identified as live (healthy + bleached) coral. In 2016 and 2017, no corals in the transects were still bleached, and were therefore identified as either live or dead. Transect survey data met assumptions for normality (Shapiro Test) and homoscedasticity (Levene’s test) and were analyzed with three-way ANOVA tests with post hoc Tukey Honest Significant Difference tests to evaluate the effect of side (west and east), depth (shallow, mid-depth, and deep), and year (2015, 2016, 2017) on live coral cover and. A two-way ANOVA with post hoc Tukey Honest Significant Difference test was used to evaluate the effect of side and depth on bleached coral cover. All statistical analyses were conducted in R (version 3.0.1). Crustose coralline algae cover and juvenile coral data were provided by the Ecosystem Sciences Division of the NOAA Pacific Islands Fisheries Science Center.

### Water sampling

Discrete seawater samples were collected during each sampling period for salinity, nutrients, total alkalinity (TA), and dissolved inorganic carbon (DIC). Temperature and depth were recorded with Seabird Electronics (SBE) 19 plus CTD profiler (March 2008- May 2012, April 2015), HOBO temperature loggers (September 2012), and Sensus Ultra dive data loggers (2015-2016). Seawater samples from March 2008, April 2010, May 2012, and April 2015 were collected during the NOAA Pacific Reef Assessment and Monitoring Program cruises, and data were provided by the NOAA Pacific Islands Fisheries Science Center, Ecosystem Sciences Division. Samples from September 2012, November 2015, and May 2016 were analyzed at Woods Hole Oceanographic Institution (WHOI). TA and DIC analyses were performed using a Versatile Instrument for the Determination of Total inorganic carbon and titration Alkalinity (Marianda Analytics and Data) and standardized using certified reference materials obtained from Andrew Dickson (Scripps Institution of Oceanography). Salinity samples were analyzed at WHOI using a Guildline autosal salinometer, and nutrient samples were run at the WHOI Nutrient Analytical Facility. Full CO_2_ system parameters were calculated from temperature, salinity, TA, and DIC using CO2SYS with the constants of Mehrbach et al.^41^ refit by Dickson and Millero^42^. Nutrient and carbonate chemistry values were consistent down to 20 m depth, and samples collected between 0 m and 20 m were averaged.

### Instrument deployments

In situ, long-term temperature logger data were provided by NOAA and were collected by SBE 39 and SBE 56 temperature loggers (Sea-bird Electronics, 5-30 min sampling interval) on the west (0.369 °S, 160.008 °W) and east (0.372 °S, 159.983 °W) sides of Jarvis. Short-term oceanographic instrument deployments were conducted at the same sites on 12–15 November 2015 and 16–23 May 2016. Instrument package deployments included a SAMI-pH sensor (Sunburst Sensors, 15 min sampling interval), SBE-37 Microcat (Sea-Bird Electronics, 20 s sampling interval), and dissolved oxygen sensor (RBR, 1 min sampling interval) which were affixed to the reef at 7 m (east) and 10 m (west) depth.

### Code Availability

The coral core analysis program coralCT is available online at https://zenodo.org/record/57855#.W5vwiPkpDz4.

## Electronic supplementary material

Supplementary Material

## Data Availability

Coral skeletal core, ecological, and oceanographic data analyzed in the current study are presented in the Supplementary Materials and are available in the BCO-DMO data collection (https://www.bco-dmo.org/project/687813). Additional long-term oceanographic data and temperature time series collected as part of the National Coral Reef Monitoring Program are available from Data.gov (http://catalog.data.gov/).
