# Supplementary material for: Repeat bleaching of a central Pacific coral reef over the past six decades (1960–2016)
Source: Commun Biol. 2018 Nov 8;1:177. doi: 10.1038/s42003-018-0183-7 (PMC6224388; doi:10.1038/s42003-018-0183-7)
Supplement: Supplementary file 1 — Supplementary Material [file 42003_2018_183_MOESM1_ESM.docx]

Supplementary Figures


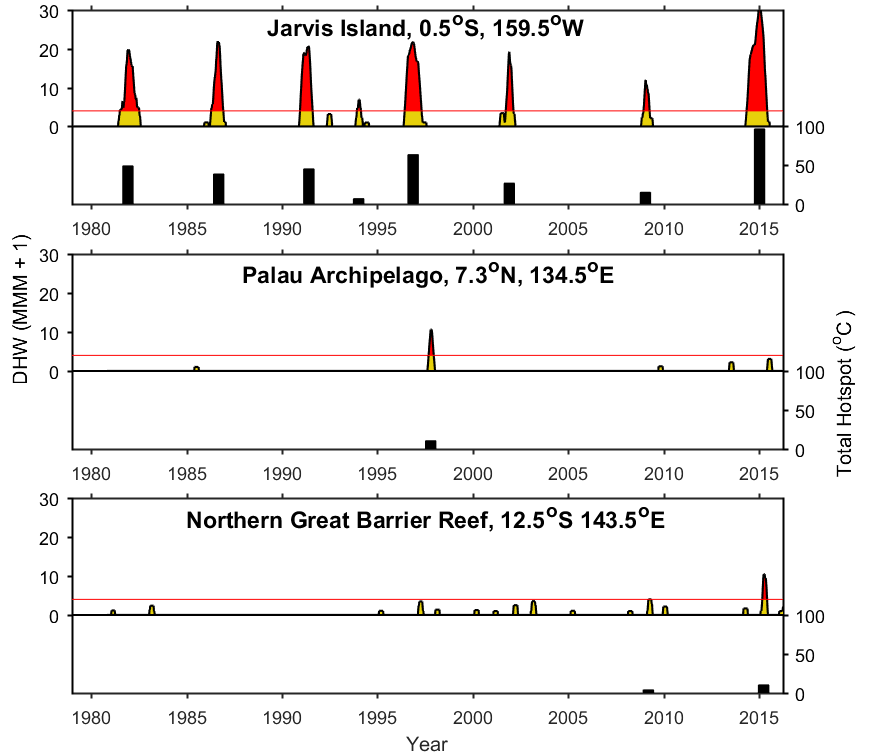


**Supplementary Figure 1.** A comparative history of thermal stress represented by the traditional NOAA Degree Heating Weeks (DHWs) calculation and cumulative DHWs or Total Hotspot on Jarvis Island (central equatorial Pacific), Palau (western tropical Pacific) and northern Great Barrier Reef since 1980. DHWs for Jarvis Island are overestimated using the traditional method compared with those calculated using the percentile method (Cf. Figure 1).

**
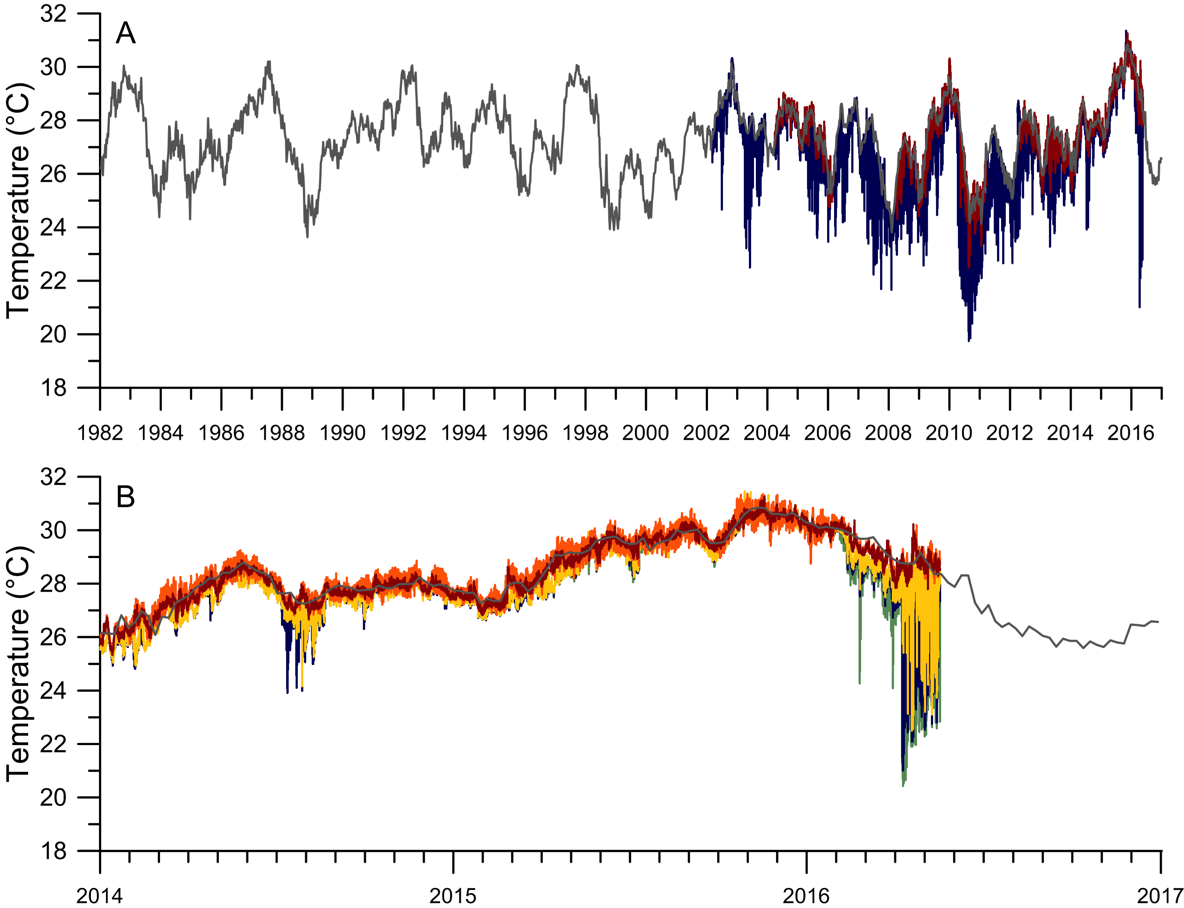
**

**Supplementary Figure 2.** Satellite and in situ temperature time series for Jarvis Island. **(A**) Temperatures at Jarvis from 1982 to 2016, collected by satellite (IGOSS Reyn_Smith OIv2, 1° x 1° resolution, gray), plotted with in situ temperature logger measurements at 12-14 m depth on the west (blue) and east (red) sides of the island from 2002-2016. (**B**) Time series of in situ temperatures from January 2014 to May 2016, collected by loggers deployed on the west (upwelling) side of the island at 5 m (yellow), 15 m (blue), and 25 m (green), and on the east side (non-upwelling) at 5 m (orange) and 15 m (red), plotted with weekly satellite data for January 2015 to December 2016 (gray).


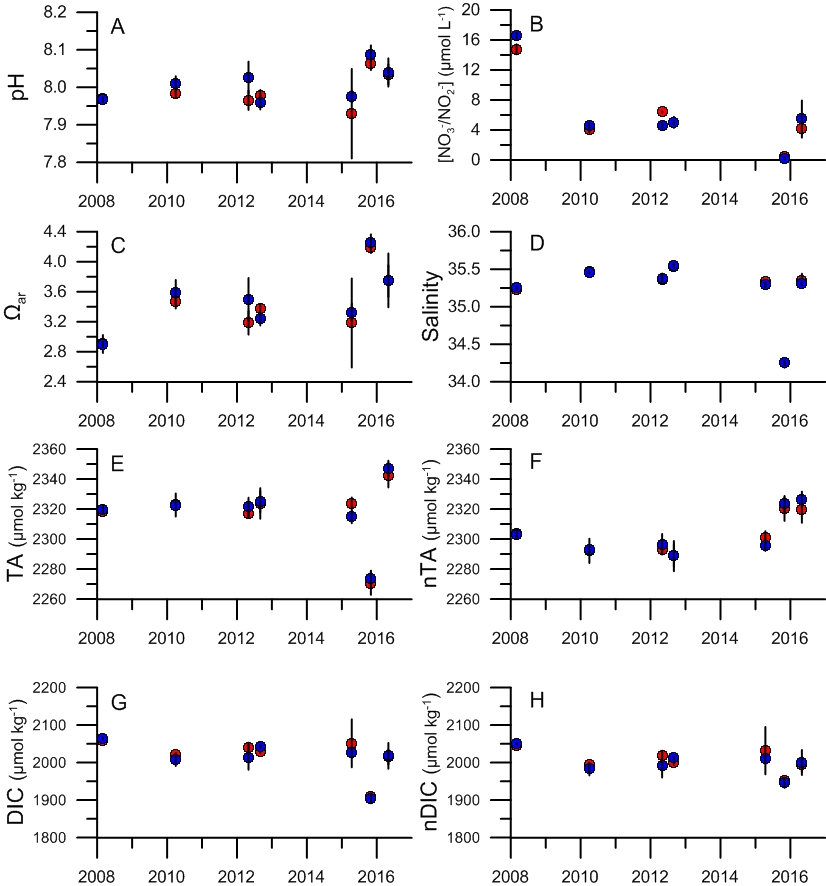


**Supplementary Figure 3.** Seawater chemistry time series for Jarvis Island, 2008-2016. Shown are mean (± one standard deviation) values for (**A**) pH (total scale), (**B**) total concentration of nitrate (NO_3_^-^) and nitrite (NO_2_^-^), (**C**) aragonite saturation state (Ω_ar_), (**D**) salinity, (**E**) total alkalinity (TA), (**F**) salinity-normalized total alkalinity (normalized to S=35), (**G**) dissolved inorganic carbon (DIC), (**H**) and salinity-normalized dissolved inorganic carbon (normalized to S=35) for the west (blue) and east (red) sides of the island.

**
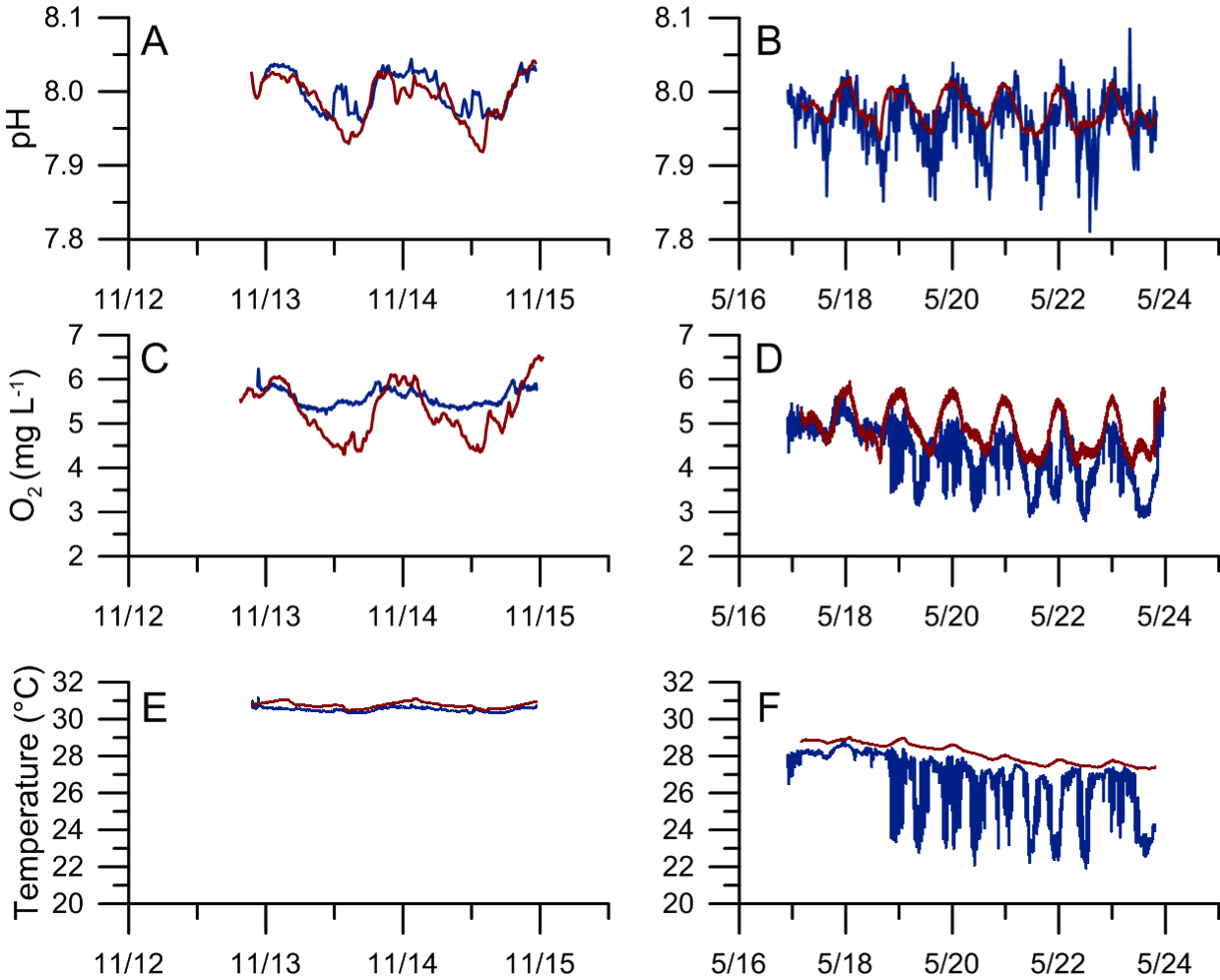
**

**Supplementary Figure 4.** Time series for pH (**A** and **B**), dissolved oxygen (**C** and **D**), and in situ temperature (**E** and **F**) collected at the height of El Niño on 12-15 November 2015 (**A**, **C**, **E**) and after severe El Niño conditions had subsided on 16-24 May 2016 (**B**, **D**, **F**) for the west (blue; upwelling) and east (red; non-upwelling) sides of Jarvis Island. Data were collected by SAMI-pH sensors (Sunburst Sensors, 15 min sampling interval), dissolved oxygen sensors (RBR, 1 min sampling interval), and SBE-37 Microcats (Sea-Bird Electronics, 20 sec sampling interval) affixed to the reef at 7-10 m depth.

**
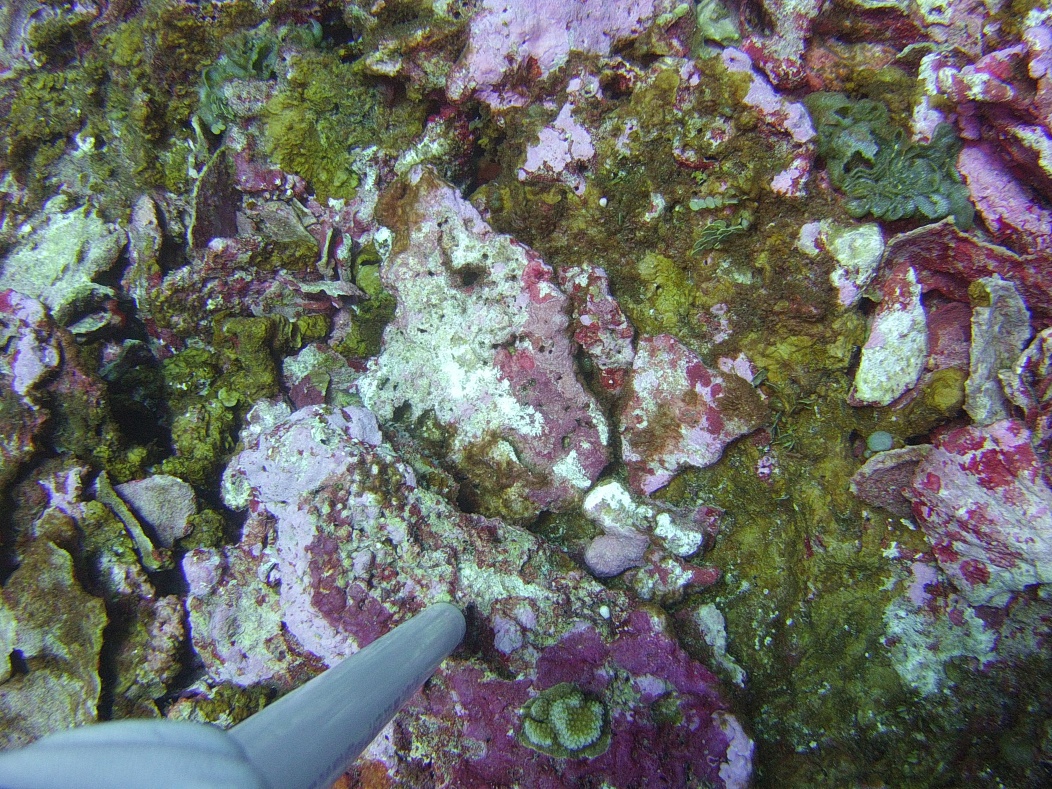
**

**
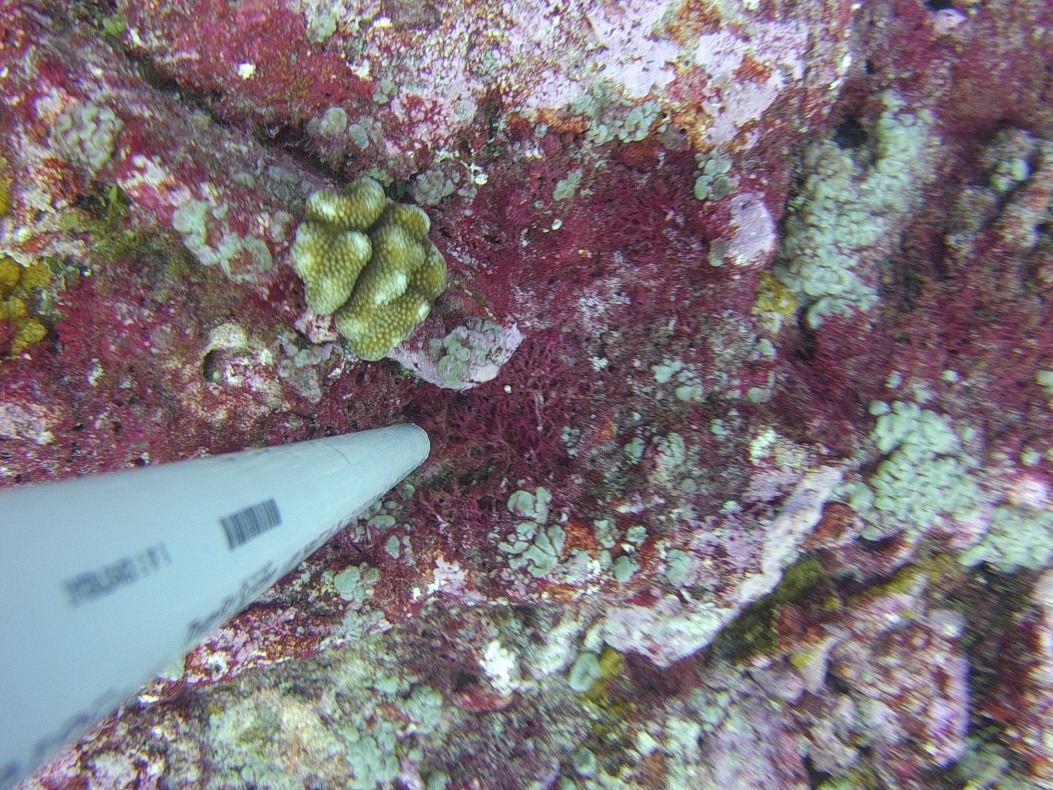
**

**Supplementary Figure 5.** Juvenile *Pocillopora* colonies on coralline algae captured in our photo transects during the 2016 and 2017 expeditions to Jarvis. For scale, the pole is 2.5 cm in diameter.

**
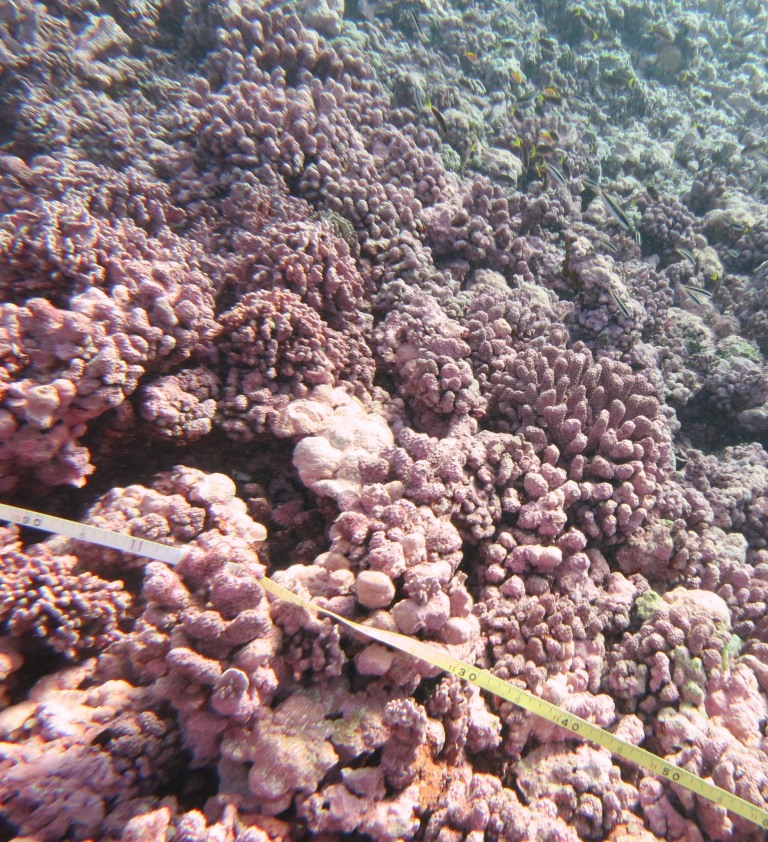
**

**Supplementary Figure 6.** Crustose coralline algae, favored settlement substrate for juvenile corals, coats the reef floor at Jarvis Island in April 2017, a little over a year after a catastrophic bleaching event killed 95% of the coral community. In this photo, recently dead corals can be seen encrusted with crustose several millimeters thick.

**
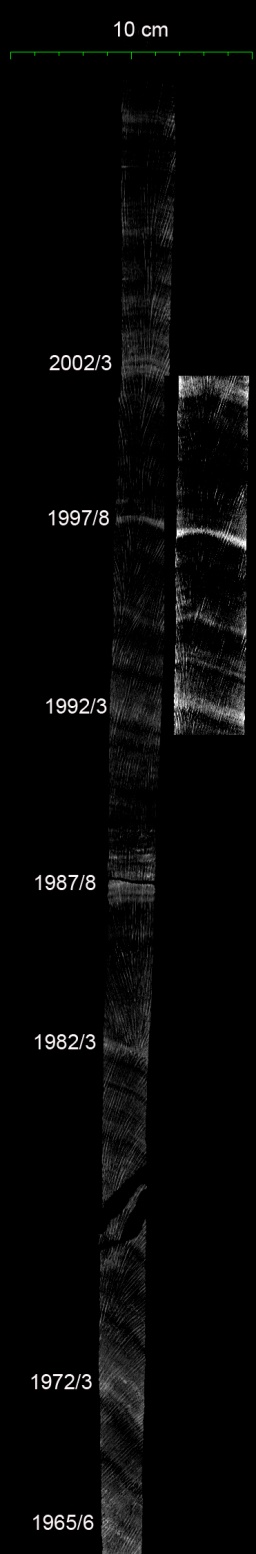
**

**Supplementary Figure 7.** CT scan of core from *Porites* ID #500 collected in September 2012 from the east side of Jarvis Island (0.371°S, 159.982°W) (Supplementary Tables 1 and 2). The image contrast is optimized to reveal the stress bands for visualization purposes. Stress bands can appear stark (e.g., inset) or less stark, depending on the baseline skeletal density upon which the stress band is superimposed. However, stress bands are not identified by eye. We utilize an automated code written in MATLAB that identifies stress bands as statistically significantly different (greater than twice the standard deviation) from the detrended whole core density (Figure 3). While conservative, this method obviates the subjectivity of stress band ID and standardizes the analysis across all cores (scale bar = 10cm).


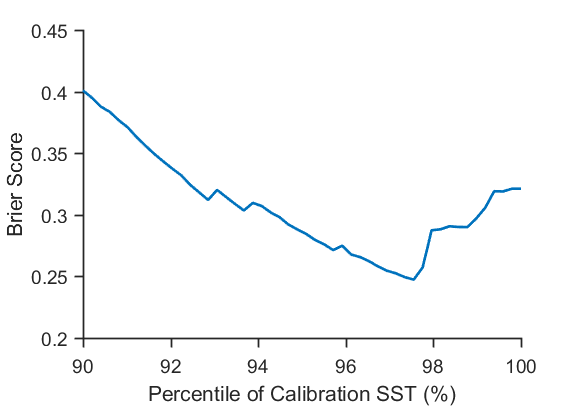


**Supplementary Figure 8.** Distribution of Brier score for each percentile of weekly SSTs over the ENSO neutral calibration period (1984-5, 1990, 1993, 1996) used to calculate bleaching thresholds for reefs reported in Donner *et al.,* (2017)^1^. The percentile used to set the thermal threshold at each site was varied across a range of 90.00^th^ to 99.99^th^ at increments of 0.01. The minimum Brier score, and therefore the maximum predictive power occurred at the 97.55^th^ percentile.

**Supplementary Tables**

**Supplementary Table 1**. Average (one standard error) percent live coral cover and bleaching on the west and east sides of Jarvis Island calculated from three replicate transect surveys at shallow (5-14 m), mid (15-19 m), and deep (20-25 m) reef sites at the height of El Niño in November 2015 and following the bleaching event in May 2016 and April 2017. No corals were bleached in 2016 or 2017.

| **Date** | **Side** | **Depth** | **% Coral Cover (SE)** | **% Bleached (SE)** |
| --- | --- | --- | --- | --- |
| Nov 2015 | West | Shallow | 9.3 (2.8) | 100.0 (0.0) |
|  |  | Mid | 33.1 (3.9) | 99.4 (0.3) |
|  |  | Deep | 29.1 (3.1) | 83.8 (7.7) |
|  | East | Shallow | 16.3 (2.5) | 99.7 (0.3) |
|  |  | Mid | 31.9 (5.2) | 96.7 (1.5) |
|  |  | Deep | 31.8 (1.6) | 92.5 (1.6) |
| May 2016 | West | Shallow | 0.0 (0.0) | - |
|  |  | Mid | 1.3 (1.0) | - |
|  |  | Deep | 4.4 (0.6) | - |
|  | East | Shallow | 4.4 (2.2) | - |
|  |  | Mid | 0.0 (0.0) | - |
|  |  | Deep | 0.1 (0.2) | - |
| Apr 2017 | West | Shallow | 0.5 (0.5) | - |
|  |  | Mid | 2.3 (1.1) | - |
|  |  | Deep | 6.3 (1.3) | - |
|  | East | Shallow | 1.9 (0.2) | - |
|  |  | Mid | 1.0 (0.1) | - |
|  |  | Deep | 0.6 (0.3) | - |

**Supplementary Table 2.** ANOVA results for a three-way test on live coral cover and two-way test on bleached coral cover.

| **Variable** | **df** | **SS** | **MS** | **F-value** | **p-value** |
| --- | --- | --- | --- | --- | --- |
| *Live coral cover* | |  |  |  |  |
| Depth | 2 | 497 | 249 | 7.39 | 0.002 |
| Year | 1 | 6557 | 3278 | 97.43 | < 0.001 |
| Side | 1 | 0 | 0 | 0.01 | 0.91 |
| Residuals | 48 | 1615 | 34 |  |  |
| *Bleached cover* | |  |  |  |  |
| Depth | 2 | 472.5 | 236.3 | 6.64 | 0.009 |
| Side | 1 | 16.1 | 16.1 | 0.45 | 0.51 |
| Residuals | 14 | 498.5 | 35.6 |  |  |

**Supplementary Table 3.** Seawater carbonate chemistry data for Jarvis Island. Mean (one standard deviation) values for temperature, salinity, total concentration of nitrate (NO_3_^-^) and nitrite (NO_2_^-^), pH (total scale), aragonite saturation state (Ω_ar_), total alkalinity (TA), and dissolved inorganic carbon (DIC) measured on the west and east sides of Jarvis during six field expeditions between 2008 and 2016. Data from March 2008, April 2010, May 2012, and April 2015 were provided by the NOAA Pacific Islands Fisheries Science Center, Ecosystem Sciences Division.

| Date | Side | n | Temp  (ºC) | Salinity  (psu) | [NO_3/2_^-^]  (μM) | pH | Ω_ar_ | TA  (μmol kg^-1^) | DIC  (μmol kg^-1^) |
| --- | --- | --- | --- | --- | --- | --- | --- | --- | --- |
| Mar 2008 | West | 16 | 24.3  (0.1) | 35.3  (0.0) | 16.6  (0.4) | 7.97  (0.01) | 2.9  (0.0) | 2320  (3) | 2065  (3) |
|  | East | 6 | 24.8  (0.1) | 35.2  (0.0) | 14.8  (0.7) | 7.97  (0.00) | 2.9  (0.1) | 2318  (2) | 2058  (2) |
| Apr  2010 | West | 4 | 28.4  (0.3) | 35.5  (0.0) | 4.6  (0.4) | 8.01  (0.02) | 3.6  (0.2) | 2322  (2) | 2008  (17) |
|  | East | 6 | 28.6  (0.1) | 35.5  (0.0) | 4.0  (0.4) | 7.98  (0.01) | 3.5  (0.1) | 2323  (8) | 2022  (2) |
| May 2012 | West | 5 | 26.6  (0.4) | 35.4  (0.1) | 5.9  (0.3) | 8.03  (0.04) | 3.5  (0.3) | 2321  (6) | 2014  (33) |
|  | East | 4 | 27.1  (0.1) | 35.4  (0.0) | 6.4  (0.9) | 7.97  (0.03) | 3.2  (0.2) | 2317  (3) | 2014  (13) |
| Sep  2012 | West | 4 | 27.8  (0.5) | 35.5  (0.0) | 5.0  (0.3) | 7.96  (0.02) | 3.2  (0.1) | 2325  (7) | 2044  (5) |
|  | East | 3 | 28.0  (0.4) | 35.5  (0.0) | 5.0  (0.7) | 7.98  (0.01) | 3.4  (0.1) | 2323  (10) | 2030  (5) |
| Apr 2015 | West | 7 | 27.9  (0.2) | 35.3  (0.0) | *No data* | 7.98  (0.02) | 3.3  (0.1) | 2315  (4) | 2027  (14) |
|  | East | 6 | 28.6  (0.1) | 35.3  (0.0) | *No data* | 7.93  (0.12) | 3.2  (0.6) | 2323  (4) | 2052  (64) |
| Nov 2015 | West | 23 | 30.6  (1.2) | 34.3  (0.0) | 0.2  (0.2) | 8.09  (0.03) | 4.3  (0.1) | 2274  (5) | 1904  (13) |
|  | East | 17 | 31.3  (0.7) | 34.3  (0.1) | 0.5  (0.3) | 8.06  (0.02) | 4.2  (0.1) | 2270  (8) | 1909  (9) |
| May 2016 | West | 31 | 27.7  (1.7) | 35.3  (0.0) | 5.5  (2.5) | 8.04  (0.04) | 3.8  (0.4) | 2347  (5) | 2018  (35) |
|  | East | 18 | 28.0  (0.7) | 35.4  (0.1) | 4.2  (0.7) | 8.03  (0.03) | 3.7  (0.2) | 2343  (8) | 2015  (21) |

| **Genus** | **Density**  **(recruits m^-2^)** | **SE** |
| --- | --- | --- |
| *Acanthastrea* | 0.00 | 0.00 |
| *Acropora* | 0.00 | 0.00 |
| *Astreopora* | 0.00 | 0.00 |
| *Coscinaraea* | 0.00 | 0.00 |
| *Cycloseris* | 0.00 | 0.00 |
| *Cyphastrea* | 0.00 | 0.00 |
| *Echinophyllia* | 0.00 | 0.00 |
| *Echinopora* | 0.00 | 0.00 |
| *Favia* | 0.00 | 0.00 |
| *Favites* | 0.00 | 0.00 |
| *Fungia* | 0.21 | 0.12 |
| *Galaxea* | 0.00 | 0.00 |
| *Goniastrea* | 0.00 | 0.00 |
| *Heliopora* | 0.00 | 0.00 |
| *Hydnophora* | 0.00 | 0.00 |
| *Leptastrea* | 0.01 | 0.01 |
| *Leptoseris* | 0.00 | 0.00 |
| *Millepora* | 0.00 | 0.00 |
| *Montastrea* | 0.00 | 0.00 |
| *Montipora* | 0.00 | 0.00 |
| *Pavona* | 0.09 | 0.03 |
| *Platygyra* | 0.00 | 0.00 |
| *Plesiastrea* | 0.00 | 0.00 |
| *Pocillopora* | 0.02 | 0.01 |
| *Porites* | 0.02 | 0.01 |
| *Psammocora* | 0.01 | 0.01 |
| *Scapophyllia* | 0.00 | 0.00 |
| *Stylophora* | 0.00 | 0.00 |
| *Tubastrea* | 0.64 | 0.23 |
| *Turbinaria* | 0.01 | 0.01 |

**Supplementary Table 4.** Jarvis coral recruitment data from 2017, presented as number of recruits observed in a 1 m^2^ quadrat (n = 32). Data were provided by the NOAA Pacific Islands Fisheries Science Center, Ecosystem Sciences Division.

**Supplementary Table 5.** Mean and one standard error percent cover of crustose coralline algae observed at Jarvis between 2015 and 2017. Data were provided by the NOAA Pacific Islands Fisheries Science Center, Ecosystem Sciences Division.

| Year | Mean Cover (%) | SE Cover (%) | # sites |
| --- | --- | --- | --- |
| 2015 | 26.1 | 1.7 | 100 |
| 2016 | 17.9 | 1.6 | 60 |
| 2017 | 22.0 | 2.6 | 60 |

**Supplementary Table 6.**  Coral core identification number, collection date, side of island, latitude and longitude coordinates, and collection depth for 30 *Porites* corals from Jarvis Island that were used in the stress band analysis. Cores were collected in 2017 (Figures 3C and 4D) but were not included in the analysis. Cf. Supplementary Table 7.

| **Core ID** | **Date Collected** | **Side** | **Latitude (ºS)** | **Longitude (ºW)** | **Depth (m)** |
| --- | --- | --- | --- | --- | --- |
| JAR1-5 | Apr 2010 | West | 0.369 | 160.008 | 16.8 |
| JAR3-7 | Apr 2010 | West | 0.369 | 160.008 | 12.8 |
| JAR5-9 | Apr 2010 | West | 0.376 | 160.014 | 7.0 |
| JAR6-10 | Apr 2010 | West | 0.376 | 160.014 | 13.7 |
| JAR-P-016 | May 2012 | East | 0.374 | 159.983 | 4.6 |
| JAR-H-018 | May 2012 | East | 0.369 | 159.983 | 3.4 |
| JAR-H-017 | May 2012 | West | 0.370 | 160.008 | 11.0 |
| 499 | Sep 2012 | East | 0.371 | 159.982 | 6.1 |
| 500 | Sep 2012 | East | 0.371 | 159.982 | 5.2 |
| 481 | Sep 2012 | East | 0.371 | 159.984 | 5.2 |
| 494 | Sep 2012 | West | 0.369 | 160.008 | 11.9 |
| 490 | Sep 2012 | West | 0.370 | 160.008 | 7.3 |
| 497 | Sep 2012 | West | 0.369 | 160.008 | 16.5 |
| 1200 | Nov 2015 | East | 0.371 | 159.982 | 6.1 |
| 1201 | Nov 2015 | East | 0.371 | 159.982 | 6.1 |
| 1202 | Nov 2015 | East | 0.371 | 159.982 | 6.1 |
| 1203 | Nov 2015 | East | 0.371 | 159.982 | 6.1 |
| 1204 | Nov 2015 | East | 0.371 | 159.982 | 6.1 |
| 1205 | Nov 2015 | East | 0.371 | 159.982 | 6.1 |
| 1206 | Nov 2015 | West | 0.369 | 160.008 | 11.0 |
| 1207 | Nov 2015 | West | 0.369 | 160.008 | 9.8 |
| 1208 | Nov 2015 | West | 0.369 | 160.008 | 11.0 |
| 1209 | Nov 2015 | West | 0.369 | 160.008 | 7.9 |
| 1210 | Nov 2015 | West | 0.369 | 160.008 | 7.6 |
| 1211 | Nov 2015 | West | 0.369 | 160.008 | 7.9 |
| 1212 | Nov 2015 | West | 0.369 | 160.008 | 4.0 |
| 1213 | Nov 2015 | West | 0.369 | 160.008 | 5.2 |
| 1214 | Nov 2015 | West | 0.369 | 160.008 | 7.6 |
| 1215 | Nov 2015 | West | 0.369 | 160.008 | 4.3 |
| 1217 | May 2016 | East | 0.371 | 159.984 | 6.1 |

**Supplementary Table 7.**  Stress band presence data for 30 *Porites* corals from Jarvis Island. “×” indicates that a stress band was present in response to warming in a given year, “-“ indicates no stress band, and *blank* indicates that coral core record did not extend into that year. Note: Core removed from bleached corals in 2015 (ID 1200-1215) were short due to permitting requirements, with most containing 5 years of growth only. Therefore, no stress band were identified in these cores prior to 2015. Long core 1217 was extracted in 2016. Cf. Supplementary Table 6.

| **Core ID** | **1965-66** | **1972-73** | **1975-76** | **1982-83** | **1987-88** | **1992-93** | **1997-98** | **2002-03** | **2009-10** | **2015-16** |
| --- | --- | --- | --- | --- | --- | --- | --- | --- | --- | --- |
| JAR1-5 |  |  |  |  |  |  | × | × | × |  |
| JAR3-7 |  |  |  |  |  | × | × | - | - |  |
| JAR5-9 |  |  | - | - | × | × | × | × | - |  |
| JAR6-10 |  |  | - | - |  |  | × | × | × |  |
| JAR-P-016 | × | - | - | × | - | - | × | - | - |  |
| JAR-H-018 | - | × | - | - | × | - | × | - | - |  |
| JAR-H-017 | × | - | - | × | - | - | - | - | - |  |
| 499 |  | - | × | × | × | × | × | × | - |  |
| 500 | × | × | - | × | × | × | × | × | - |  |
| 481 | × | - | - | × | - | - | - | × | - |  |
| 494 |  | - | - | × | - | - | × | - | - |  |
| 490 | - | - | × | - | - | - | - | - | - |  |
| 497 | × | × | - | × | × | × | × | - | - |  |
| 1217 |  |  |  | - | - | × | × | - | - | × |
| 1200 |  |  |  |  |  |  |  |  |  | × |
| 1201 |  |  |  |  |  |  |  |  |  | × |
| 1202 |  |  |  |  |  |  |  |  |  | × |
| 1203 |  |  |  |  |  |  |  |  |  | × |
| 1204 |  |  |  |  |  |  |  |  |  | - |
| 1205 |  |  |  |  |  |  |  |  |  | × |
| 1206 |  |  |  |  |  |  |  |  |  | × |
| 1207 |  |  |  |  |  |  |  |  |  | × |
| 1208 |  |  |  |  |  |  |  |  |  | × |
| 1209 |  |  |  |  |  |  |  |  |  | × |
| 1210 |  |  |  |  |  |  |  |  |  | - |
| 1211 |  |  |  |  |  |  |  |  |  | × |
| 1212 |  |  |  |  |  |  |  |  |  | × |
| 1213 |  |  |  |  |  |  |  |  |  | × |
| 1214 |  |  |  |  |  |  |  |  |  | × |
| 1215 |  |  |  |  |  |  |  |  |  | × |

**Supplementary Table 8.** Comparison of predictive power of bleaching predictions made using the percentile method versus the traditional MMM method of calculating bleaching thresholds. Predictions were assessed using the bleaching observations dataset of Donner *et al.,* (2017)^1^.

| **Method** | **Bleaching Alert Level (^o^C·weeks)** | **Type 1 prediction Error (%)** | **Type 2 prediction Error (%)** | **Correct Predictions (%)** | **Brier Score (0-1) (0 desired)** |
| --- | --- | --- | --- | --- | --- |
| MMM (NOAA) | 4 | 7.0 | 67.6 | 32.4 | 0.31 |
| Percentile method | 4 | 8.1 | 46.4 | 53.6 | 0.24 |

**Supplementary References**

1. Donner, S.D., Rickbeil, G.J.M. & Heron, S.F. A new, high-resolution global mass coral bleaching database*. PLoS ONE* **12**, e0175490 (2017).
